# Posterior common femoral branch pseudoaneurysm: an unusual arterial complication following femoral venous access

**DOI:** 10.1259/bjrcr.20150335

**Published:** 2016-11-02

**Authors:** James Davies, James Metcalfe, Robert Ward

**Affiliations:** ^1^ Department of Vascular Surgery, Dorset County Hospital, Dorchester, UK; ^2^ Department of Clinical and Interventional Radiology, Dorset County Hospital, Dorchester, UK

## Abstract

A 70-year-old male presented with groin pain and swelling 11 days following a pulmonary vein isolation procedure via an unguided femoral venous puncture for atrial fibrillation. On the fourth visit, his haemoglobin level had dropped from 14.2 gl^−1^ to 10.7g l^−1^. Repeat duplex imaging revealed a large haematoma with deep flow. A CT angiogram revealed a pseudoaneurysm of a right common femoral branch artery. A subsequent angiogram revealed active bleeding, and the feeding artery was coiled. Pseudoaneurysms are recognized complications of vascular intervention, but more commonly occur anteriorly in major vessels. This elusive presentation reminds us of several important points. First, with the increasing use of interventional techniques across all medical specialties, the use of image guidance to aid vessel access is paramount for safety; not all specialties currently practise this routinely. Furthermore, we should consider arterial injury in all patients, including those who have had venous puncture. Injuries may not necessarily occur at the anterior vessel wall, and may well be deeper. Finally, there should be a low threshold for alternative imaging if symptoms are out of context with clinical findings.

## Background

Across all medical specialties, there is increasing use of minimally invasive endovascular procedures. A basic knowledge of anatomy and potential for injury in these interventions is important in order to recognize complications early. We present a case in which the diagnosis was delayed, and which provides several important learning points.

## Case

A 70-year-old male presented to the emergency department with pain and swelling in his right groin 11 days following a pulmonary vein isolation procedure with unguided femoral venous puncture for atrial fibrillation (AF). His past medical history included AF, radical prostatectomy and melanoma. He was on rivaroxaban for anticoagulation, which had been held prior to the procedure and subsequently restarted the day after the procedure.

The procedure was performed at another centre, and on two occasions afterwards, he had attended the treating centre, and twice had undergone a duplex scan of the femoral vessels that had revealed no collection or pseudoaneurysm. He attended the emergency department with severe pain and leg swelling three times, but was discharged with normal bloods and the knowledge of two negative duplex scans.

**Figure 1.**
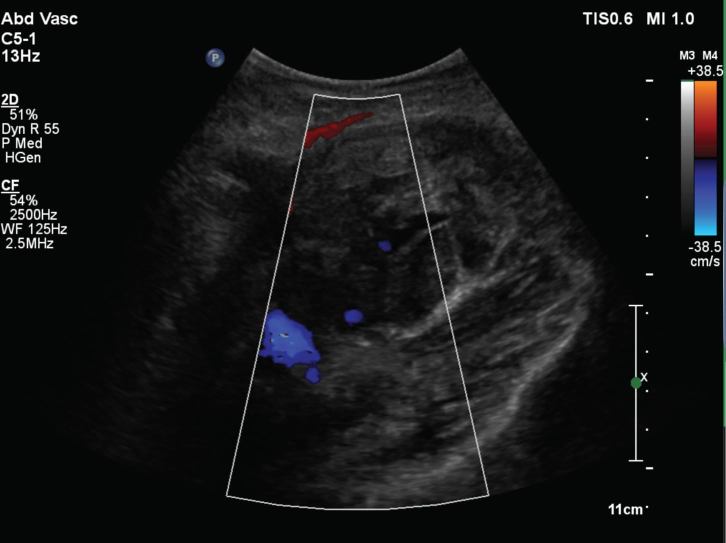
Duplex scan showing haematoma with deep flow.

**Figure 2.**
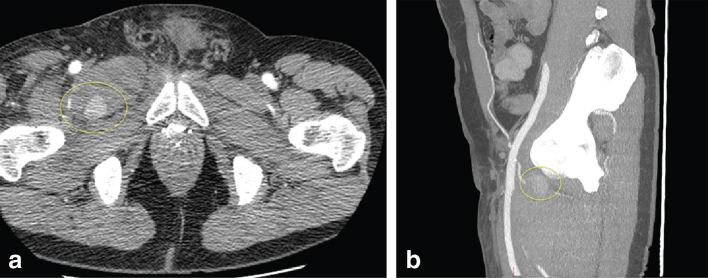
(a, b) Axial and sagittal slices of a CT angiogram showing depth of the pseudoaneurysm.

**Figure 3.**
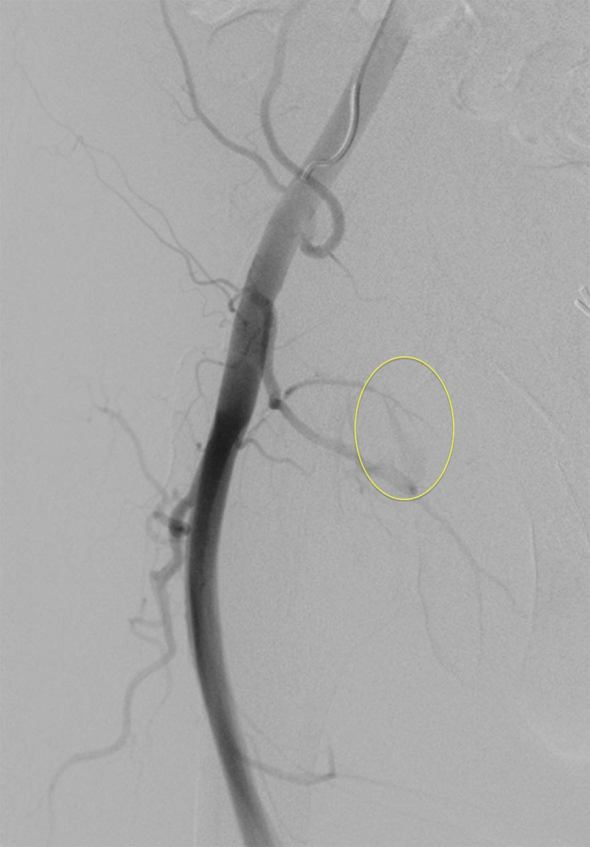
Image showing contrast blush.

**Figure 4.**
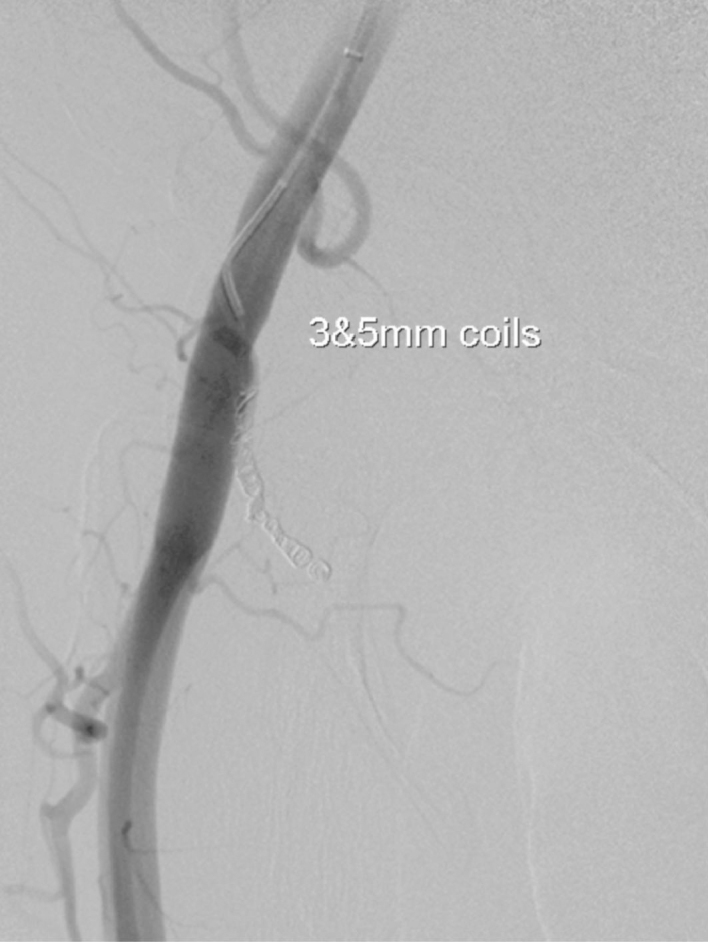
Image showing coiling of a feeding vessel.

On the fourth visit, his haemoglobin had dropped from 14.2 g l^−1^ to 10.7 g l^−1^ over 6 days. A repeat duplex in our radiology department (Figure 1) revealed a large haematoma with evidence of a deep pseudoaneurysm. A CT angiogram revealed a pseudoaneurysm of a posterior right common femoral branch artery (Figure 2a,b). A subsequent angiogram revealed active bleeding at the site (Figure 3) and the feeding artery was coiled by interventional radiology (Figure 4). The patient was admitted for observation for 24 h and discharged with no further problems. 2 months on, he remained well.

## Discussion

Pseudoaneurysms are recognized complications of vascular intervention, but more commonly occur anteriorly in the major vessels. In a retrospective study by Kacila et al,^1^ there was a pseudoaneurysm incidence of 3.7% following cardiac intervention, with an increased incidence associated with anticoagulant treatment. Banfić et al^2^ demonstrated a higher incidence of pseudoaneurysm in patients who underwent an angiogram and required intervention. Further risk factors for pseudoaneurysm formation include low femoral puncture, increased length of procedure, larger sheath size ≥ 7 French, difficult access, hypertension and simultaneous arteriovenous access.^3^ Traditionally, ultrasound has been the diagnostic modality of choice, often demonstrating turbulent flow or the to and fro sign that is considered pathagnomic and occurs as a result of diastolic reversal of flow in the aneurysm neck.

**Figure 5.**
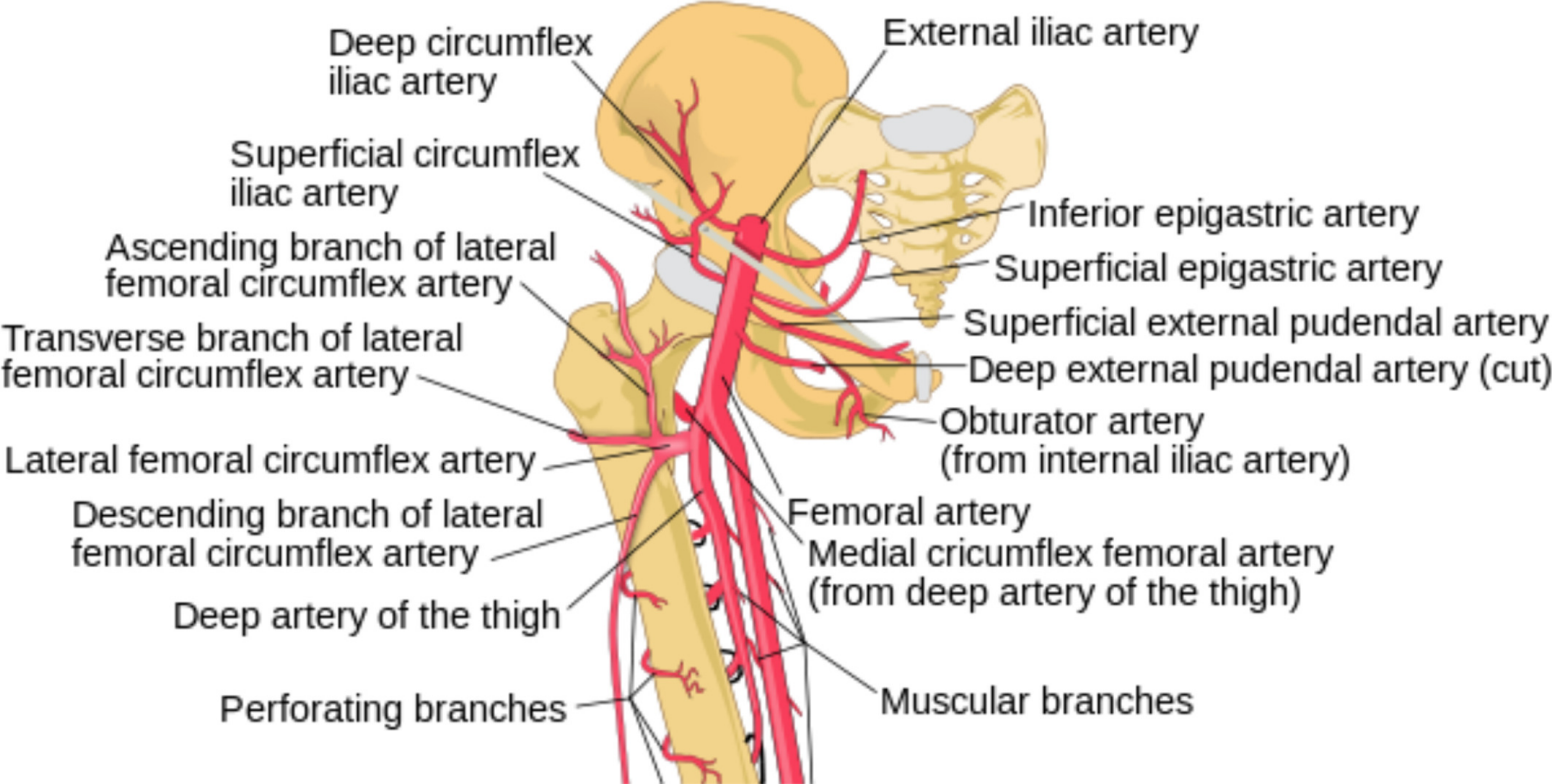
Femoral artery branches. Adapted from Ruiz Villareal^4^ published under the Creative Commons Attribution Share Alike 3.0 unported license https://creativecommons.org/licenses/by-sa/3.0/.

There are multiple branches of the femoral and profunda femoris arteries (Figure 5). Given this intricate arrangement of vessels in a relatively small space, the potential for injury is high. Lying adjacent and medial to the common femoral artery is the common femoral vein. In procedures such as this where the target vessel is the vein, the three arteries that are most at risk are the superficial epigastric, and the superficial and deep external pudendal arteries, which can be seen coursing medially off the artery in the diagram. The other vessel at risk is the medial circumflex femoral artery and its branches, as it leaves the profunda femoris medially before looping laterally to encircle the femoral neck. Injuries here are more common when the arterial puncture site is low. In our case, it is difficult to say exactly which vessel was damaged, but it is likely that it was the deep external pudendal artery or one of its branches.

This is by no means a first report of such a case. Shannon et al^5^ report a similar case, which was complicated by major retroperitoneal haemorrhage, managed by endovascular coiling. Waldherr et al^6^ report superselective embolization of a deep femoral artery branch pseudoaneurysm sustained through both percutaneous coronary intervention and as a complication of hip surgery. The management of pseudoaneurysms depends on several factors including size and location. Small, superficial aneurysms may be treated with thrombin injection, whereas larger ones may require formal surgical excision and arterial repair. In cases such as this where the bleeding vessel is deeper, the first approach should be endovascular, as it is less invasive and carries a lower morbidity.

Inspite of this injury not being a first report, this delayed presentation reminds us of several important points. First, with the increasing use of interventional techniques across all medical specialties, the use of image guidance to aid vessel access is paramount for safety; not all specialties currently practise this routinely. Furthermore, we should consider arterial injury in all patients, including those who have had venous puncture. Injuries may not necessarily occur at the anterior vessel wall, and may well be deeper. Finally, there should be a low threshold for alternative imaging if symptoms are out of context with clinical findings.

## Conclusions

We present an elusive complication of vascular access. Conventional first-line imaging techniques here can be falsely reassuring and a high suspicion of this type of complication is paramount in the face of on-going symptoms.

## Learning points

1. Interventional procedures are increasingly common across all specialties.
2. Image-guided access to vessels is the gold standard.
3. Posterior vessel wall and potentially deeper injuries may occur.
4. In the absence of positive findings on initial imaging, and ongoing symptoms, a low threshold for further cross-sectional imaging should exist.
